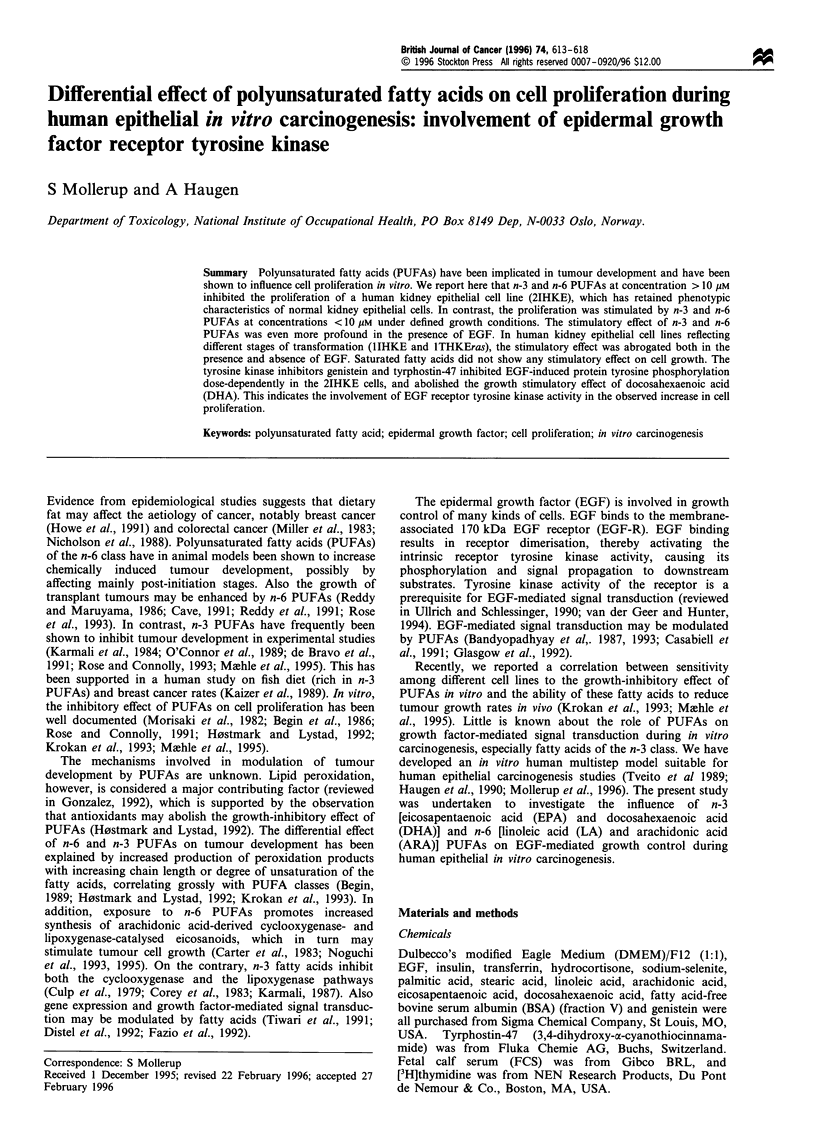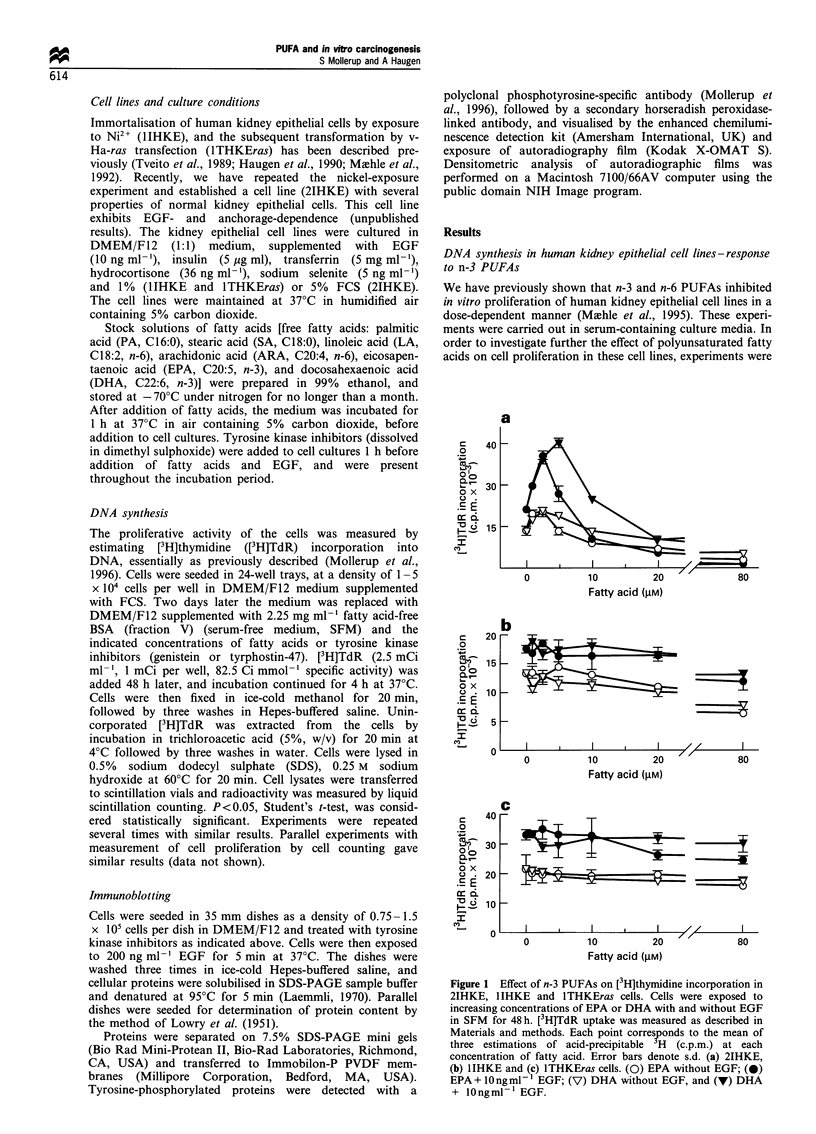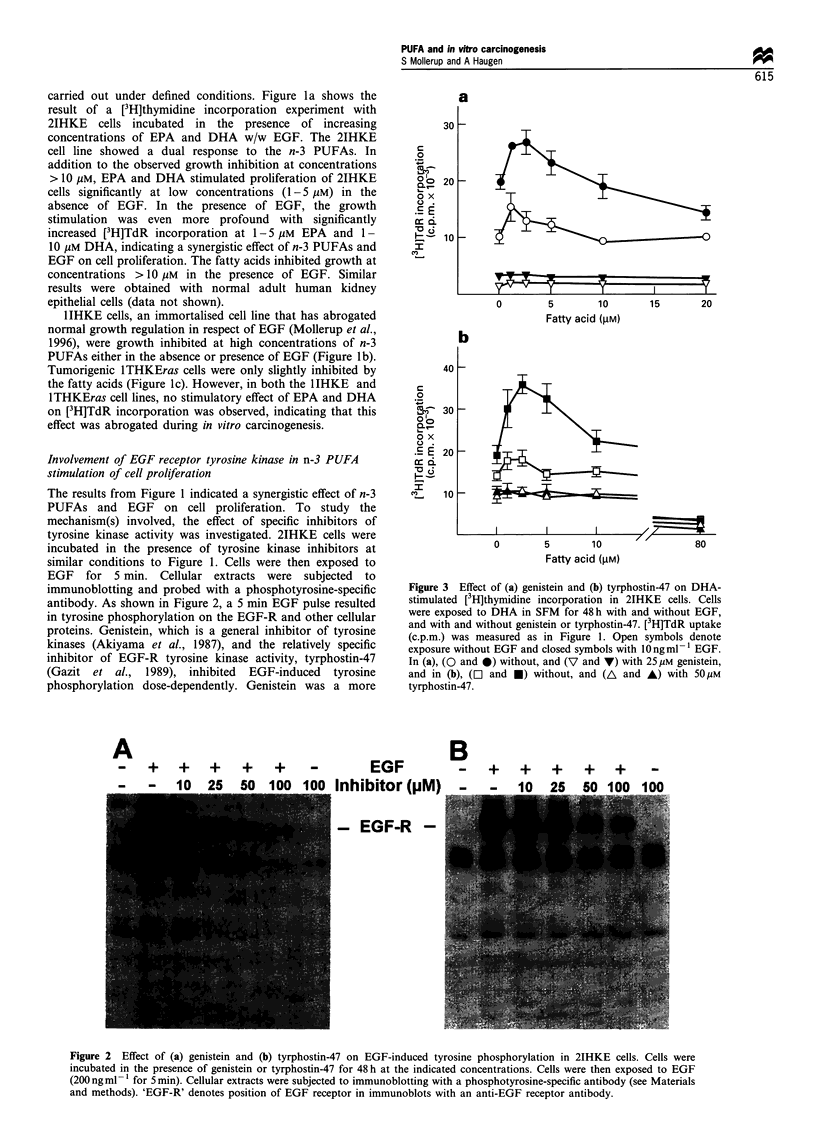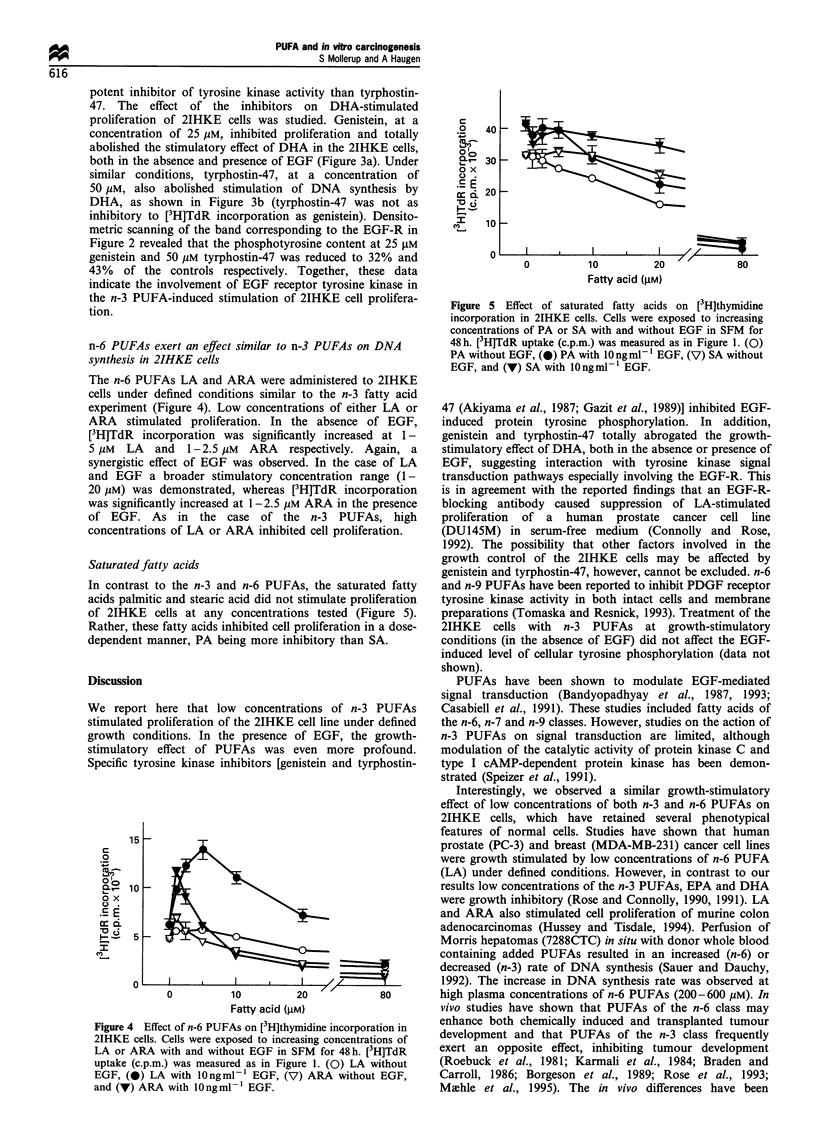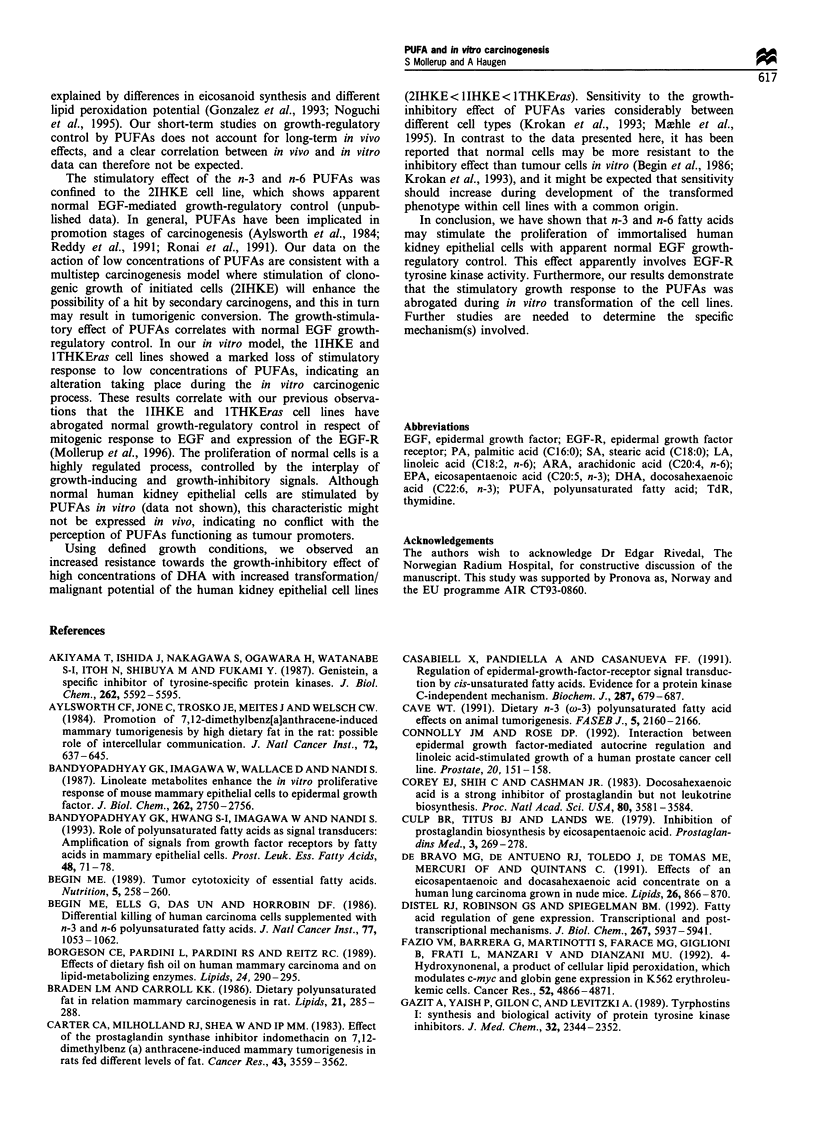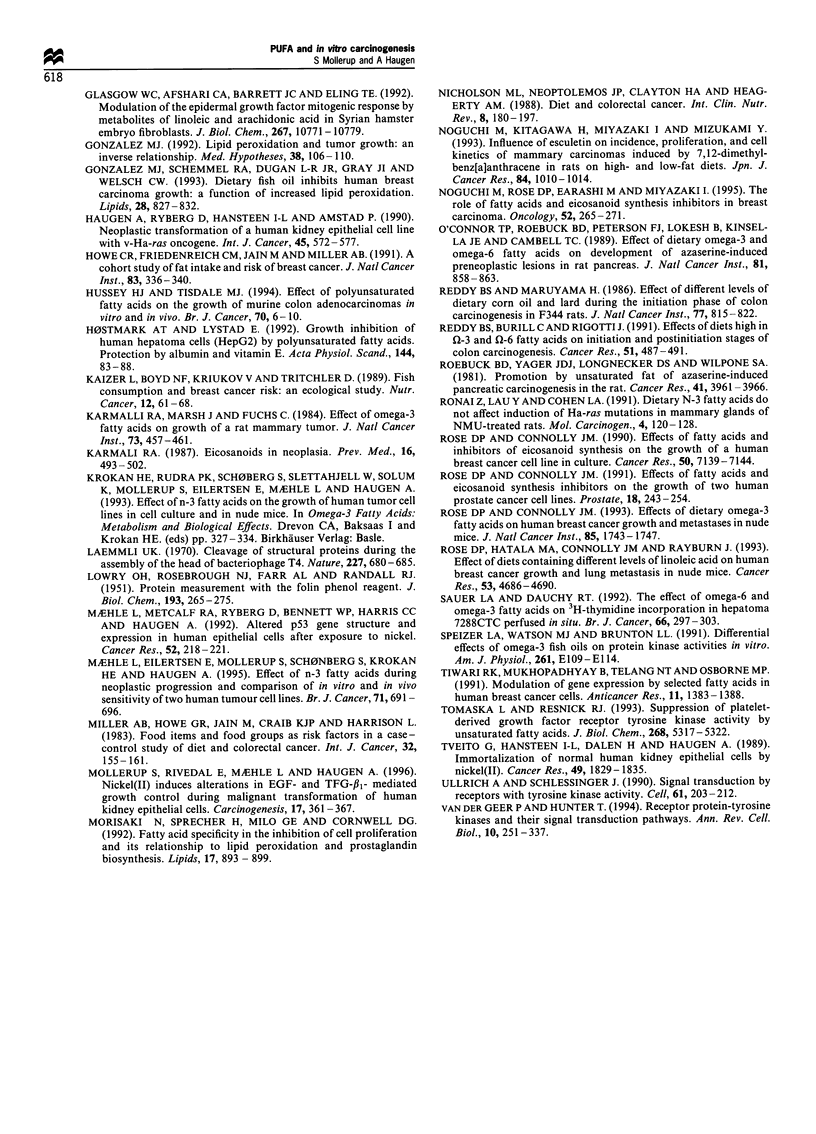# Differential effect of polyunsaturated fatty acids on cell proliferation during human epithelial in vitro carcinogenesis: involvement of epidermal growth factor receptor tyrosine kinase.

**DOI:** 10.1038/bjc.1996.410

**Published:** 1996-08

**Authors:** S. Mollerup, A. Haugen

**Affiliations:** Department of Toxicology, National Institute of Occupational Health, Oslo, Norway.

## Abstract

**Images:**


British Journal of Cancer (1996) 74, 613-618
© 1996 Stockton Press All rights reserved 0007-0920/96 $12.00

### Differential effect of polyunsaturated fatty acids on cell proliferation during human epithelial *in vitro* carcinogenesis: involvement of epidermal growth factor receptor tyrosine kinase

S Mollerup and A Haugen

*Department of Toxicology, National Institute of Occupational Health, PO Box 8149 Dep, N-0033 Oslo, Norway.*

**Summary** Polyunsaturated fatty acids (PUFAs) have been implicated in tumour development and have been shown to influence cell proliferation *in vitro*. We report here that *n*-3 and *n*-6 PUFAs at concentration >10 μM inhibited the proliferation of a human kidney epithelial cell line (2IHKE), which has retained phenotypic characteristics of normal kidney epithelial cells. In contrast, the proliferation was stimulated by *n*-3 and *n*-6 PUFAs at concentrations <10 μM under defined growth conditions. The stimulatory effect of *n*-3 and *n*-6 PUFAs was even more profound in the presence of EGF. In human kidney epithelial cell lines reflecting different stages of transformation (1IHKE and 1THKE*ras*), the stimulatory effect was abrogated both in the presence and absence of EGF. Saturated fatty acids did not show any stimulatory effect on cell growth. The tyrosine kinase inhibitors genistein and tyrphostin-47 inhibited EGF-induced protein tyrosine phosphorylation dose-dependently in the 2IHKE cells, and abolished the growth stimulatory effect of docosahexaenoic acid (DHA). This indicates the involvement of EGF receptor tyrosine kinase activity in the observed increase in cell proliferation.

**Keywords:** polyunsaturated fatty acid; epidermal growth factor; cell proliferation; *in vitro* carcinogenesis

Evidence from epidemiological studies suggests that dietary fat may affect the aetiology of cancer, notably breast cancer (Howe *et al.*, 1991) and colorectal cancer (Miller *et al.*, 1983; Nicholson *et al.*, 1988). Polyunsaturated fatty acids (PUFAs) of the *n*-6 class have in animal models been shown to increase chemically induced tumour development, possibly by affecting mainly post-initiation stages. Also the growth of transplant tumours may be enhanced by *n*-6 PUFAs (Reddy and Maruyama, 1986; Cave, 1991; Reddy *et al.*, 1991; Rose *et al.*, 1993). In contrast, *n*-3 PUFAs have frequently been shown to inhibit tumour development in experimental studies (Karmali *et al.*, 1984; O'Connor *et al.*, 1989; de Bravo *et al.*, 1991; Rose and Connolly, 1993; Mæhle *et al.*, 1995). This has been supported in a human study on fish diet (rich in *n*-3 PUFAs) and breast cancer rates (Kaizer *et al.*, 1989). *In vitro*, the inhibitory effect of PUFAs on cell proliferation has been well documented (Morisaki *et al.*, 1982; Begin *et al.*, 1986; Rose and Connolly, 1991; Høstmark and Lystad, 1992; Krokan *et al.*, 1993; Mæhle *et al.*, 1995).

The mechanisms involved in modulation of tumour development by PUFAs are unknown. Lipid peroxidation, however, is considered a major contributing factor (reviewed in Gonzalez, 1992), which is supported by the observation that antioxidants may abolish the growth-inhibitory effect of PUFAs (Høstmark and Lystad, 1992). The differential effect of *n*-6 and *n*-3 PUFAs on tumour development has been explained by increased production of peroxidation products with increasing chain length or degree of unsaturation of the fatty acids, correlating grossly with PUFA classes (Begin, 1989; Høstmark and Lystad, 1992; Krokan *et al.*, 1993). In addition, exposure to *n*-6 PUFAs promotes increased synthesis of arachidonic acid-derived cyclooxygenase- and lipoxygenase-catalysed eicosanoids, which in turn may stimulate tumour cell growth (Carter *et al.*, 1983; Noguchi *et al.*, 1993, 1995). On the contrary, *n*-3 fatty acids inhibit both the cyclooxygenase and the lipoxygenase pathways (Culp *et al.*, 1979; Corey *et al.*, 1983; Karmali, 1987). Also gene expression and growth factor-mediated signal transduction may be modulated by fatty acids (Tiwari *et al.*, 1991; Distel *et al.*, 1992; Fazio *et al.*, 1992).

Correspondence: S Mollerup
Received 1 December 1995; revised 22 February 1996; accepted 27 February 1996

The epidermal growth factor (EGF) is involved in growth control of many kinds of cells. EGF binds to the membrane-associated 170 kDa EGF receptor (EGF-R). EGF binding results in receptor dimerisation, thereby activating the intrinsic receptor tyrosine kinase activity, causing its phosphorylation and signal propagation to downstream substrates. Tyrosine kinase activity of the receptor is a prerequisite for EGF-mediated signal transduction (reviewed in Ullrich and Schlessinger, 1990; van der Geer and Hunter, 1994). EGF-mediated signal transduction may be modulated by PUFAs (Bandyopadhyay *et al*,. 1987, 1993; Casabiell *et al.*, 1991; Glasgow *et al.*, 1992).

Recently, we reported a correlation between sensitivity among different cell lines to the growth-inhibitory effect of PUFAs *in vitro* and the ability of these fatty acids to reduce tumour growth rates *in vivo* (Krokan *et al.*, 1993; Mæhle *et al.*, 1995). Little is known about the role of PUFAs on growth factor-mediated signal transduction during *in vitro* carcinogenesis, especially fatty acids of the *n*-3 class. We have developed an *in vitro* human multistep model suitable for human epithelial carcinogenesis studies (Tveito *et al* 1989; Haugen *et al.*, 1990; Mollerup *et al.*, 1996). The present study was undertaken to investigate the influence of *n*-3 [eicosapentaenoic acid (EPA) and docosahexaenoic acid (DHA)] and *n*-6 [linoleic acid (LA) and arachidonic acid (ARA)] PUFAs on EGF-mediated growth control during human epithelial *in vitro* carcinogenesis.

### Materials and methods

### *Chemicals*

Dulbecco's modified Eagle Medium (DMEM)/F12 (1:1), EGF, insulin, transferrin, hydrocortisone, sodium-selenite, palmitic acid, stearic acid, linoleic acid, arachidonic acid, eicosapentaenoic acid, docosahexaenoic acid, fatty acid-free bovine serum albumin (BSA) (fraction V) and genistein were all purchased from Sigma Chemical Company, St Louis, MO, USA. Tyrphostin-47 (3,4-dihydroxy-α-cyanothiocinnamamide) was from Fluka Chemie AG, Buchs, Switzerland. Fetal calf serum (FCS) was from Gibco BRL, and [$^{3}$H]thymidine was from NEN Research Products, Du Pont de Nemour & Co., Boston, MA, USA.

### *Cell lines and culture conditions*

Immortalisation of human kidney epithelial cells by exposure to $Ni^{2+}$ (1IHKE), and the subsequent transformation by v-Ha-*ras* transfection (1THKE*ras*) has been described previously (Tveito *et al.*, 1989; Haugen *et al.*, 1990; Mæhle *et al.*, 1992). Recently, we have repeated the nickel-exposure experiment and established a cell line (2IHKE) with several properties of normal kidney epithelial cells. This cell line exhibits EGF- and anchorage-dependence (unpublished results). The kidney epithelial cell lines were cultured in DMEM/F12 (1:1) medium, supplemented with EGF (10 ng $ml^{-1}$), insulin (5 $\mu$g ml), transferrin (5 mg $ml^{-1}$), hydrocortisone (36 ng $ml^{-1}$), sodium selenite (5 ng $ml^{-1}$) and 1% (1IHKE and 1THKE*ras*) or 5% FCS (2IHKE). The cell lines were maintained at 37°C in humidified air containing 5% carbon dioxide.

Stock solutions of fatty acids [free fatty acids: palmitic acid (PA, C16:0), stearic acid (SA, C18:0), linoleic acid (LA, C18:2, *n*-6), arachidonic acid (ARA, C20:4, *n*-6), eicosapentaenoic acid (EPA, C20:5, *n*-3), and docosahexaenoic acid (DHA, C22:6, *n*-3)] were prepared in 99% ethanol, and stored at −70°C under nitrogen for no longer than a month. After addition of fatty acids, the medium was incubated for 1 h at 37°C in air containing 5% carbon dioxide, before addition to cell cultures. Tyrosine kinase inhibitors (dissolved in dimethyl sulphoxide) were added to cell cultures 1 h before addition of fatty acids and EGF, and were present throughout the incubation period.

### *DNA synthesis*

The proliferative activity of the cells was measured by estimating [$^3$H]thymidine ([$^3$H]TdR) incorporation into DNA, essentially as previously described (Mollerup *et al.*, 1996). Cells were seeded in 24-well trays, at a density of 1−5 $\times 10^4$ cells per well in DMEM/F12 medium supplemented with FCS. Two days later the medium was replaced with DMEM/F12 supplemented with 2.25 mg $ml^{-1}$ fatty acid-free BSA (fraction V) (serum-free medium, SFM) and the indicated concentrations of fatty acids or tyrosine kinase inhibitors (genistein or tyrphostin-47). [$^3$H]TdR (2.5 mCi $ml^{-1}$, 1 mCi per well, 82.5 Ci $mmol^{-1}$ specific activity) was added 48 h later, and incubation continued for 4 h at 37°C. Cells were then fixed in ice-cold methanol for 20 min, followed by three washes in Hepes-buffered saline. Unincorporated [$^3$H]TdR was extracted from the cells by incubation in trichloroacetic acid (5%, w/v) for 20 min at 4°C followed by three washes in water. Cells were lysed in 0.5% sodium dodecyl sulphate (SDS), 0.25 M sodium hydroxide at 60°C for 20 min. Cell lysates were transferred to scintillation vials and radioactivity was measured by liquid scintillation counting. $P<0.05$, Student's *t*-test, was considered statistically significant. Experiments were repeated several times with similar results. Parallel experiments with measurement of cell proliferation by cell counting gave similar results (data not shown).

### *Immunoblotting*

Cells were seeded in 35 mm dishes as a density of 0.75−1.5 $\times$ $10^5$ cells per dish in DMEM/F12 and treated with tyrosine kinase inhibitors as indicated above. Cells were then exposed to 200 ng $ml^{-1}$ EGF for 5 min at 37°C. The dishes were washed three times in ice-cold Hepes-buffered saline, and cellular proteins were solubilised in SDS-PAGE sample buffer and denatured at 95°C for 5 min (Laemmli, 1970). Parallel dishes were seeded for determination of protein content by the method of Lowry *et al.* (1951).

Proteins were separated on 7.5% SDS-PAGE mini gels (Bio Rad Mini-Protean II, Bio-Rad Laboratories, Richmond, CA, USA) and transferred to Immobilon-P PVDF membranes (Millipore Corporation, Bedford, MA, USA). Tyrosine-phosphorylated proteins were detected with a polyclonal phosphotyrosine-specific antibody (Mollerup *et al.*, 1996), followed by a secondary horseradish peroxidase-linked antibody, and visualised by the enhanced chemiluminescence detection kit (Amersham International, UK) and exposure of autoradiography film (Kodak X-OMAT S). Densitometric analysis of autoradiographic films was performed on a Macintosh 7100/66AV computer using the public domain NIH Image program.

### Results

### *DNA synthesis in human kidney epithelial cell lines–response to* n*-3 PUFAs*

We have previously shown that *n*-3 and *n*-6 PUFAs inhibited *in vitro* proliferation of human kidney epithelial cell lines in a dose-dependent manner (Mæhle *et al.*, 1995). These experiments were carried out in serum-containing culture media. In order to investigate further the effect of polyunsaturated fatty acids on cell proliferation in these cell lines, experiments were

**Figure 1** Effect of *n*-3 PUFAs on [$^3$H]thymidine incorporation in 2IHKE, 1IHKE and 1THKE*ras* cells. Cells were exposed to increasing concentrations of EPA or DHA with and without EGF in SFM for 48 h. [$^3$H]TdR uptake was measured as described in Materials and methods. Each point corresponds to the mean of three estimations of acid-precipitable $^3$H (c.p.m.) at each concentration of fatty acid. Error bars denote s.d. (**a**) 2IHKE, (**b**) 1IHKE and (**c**) 1THKE*ras* cells. (○) EPA without EGF; (●) EPA + 10 ng $ml^{-1}$ EGF; (▽) DHA without EGF, and (▼) DHA + 10 ng $ml^{-1}$ EGF.

carried out under defined conditions. Figure 1a shows the result of a [$^3$H]thymidine incorporation experiment with 2IHKE cells incubated in the presence of increasing concentrations of EPA and DHA w/w EGF. The 2IHKE cell line showed a dual response to the *n*-3 PUFAs. In addition to the observed growth inhibition at concentrations >10 $\mu$M, EPA and DHA stimulated proliferation of 2IHKE cells significantly at low concentrations (1–5 $\mu$M) in the absence of EGF. In the presence of EGF, the growth stimulation was even more profound with significantly increased [$^3$H]TdR incorporation at 1–5 $\mu$M EPA and 1–10 $\mu$M DHA, indicating a synergistic effect of *n*-3 PUFAs and EGF on cell proliferation. The fatty acids inhibited growth at concentrations >10 $\mu$M in the presence of EGF. Similar results were obtained with normal adult human kidney epithelial cells (data not shown).

1IHKE cells, an immortalised cell line that has abrogated normal growth regulation in respect of EGF (Mollerup *et al.*, 1996), were growth inhibited at high concentrations of *n*-3 PUFAs either in the absence or presence of EGF (Figure 1b). Tumorigenic 1THKE*ras* cells were only slightly inhibited by the fatty acids (Figure 1c). However, in both the 1IHKE and 1THKE*ras* cell lines, no stimulatory effect of EPA and DHA on [$^3$H]TdR incorporation was observed, indicating that this effect was abrogated during *in vitro* carcinogenesis.

### *Involvement of EGF receptor tyrosine kinase in* n-*3 PUFA stimulation of cell proliferation*

The results from Figure 1 indicated a synergistic effect of *n*-3 PUFAs and EGF on cell proliferation. To study the mechanism(s) involved, the effect of specific inhibitors of tyrosine kinase activity was investigated. 2IHKE cells were incubated in the presence of tyrosine kinase inhibitors at similar conditions to Figure 1. Cells were then exposed to EGF for 5 min. Cellular extracts were subjected to immunoblotting and probed with a phosphotyrosine-specific antibody. As shown in Figure 2, a 5 min EGF pulse resulted in tyrosine phosphorylation on the EGF-R and other cellular proteins. Genistein, which is a general inhibitor of tyrosine kinases (Akiyama *et al.*, 1987), and the relatively specific inhibitor of EGF-R tyrosine kinase activity, tyrphostin-47 (Gazit *et al.*, 1989), inhibited EGF-induced tyrosine phosphorylation dose-dependently. Genistein was a more

**Figure 3** Effect of (**a**) genistein and (**b**) tyrphostin-47 on DHA-stimulated [$^3$H]thymidine incorporation in 2IHKE cells. Cells were exposed to DHA in SFM for 48 h with and without EGF, and with and without genistein or tyrphostin-47. [$^3$H]TdR uptake (c.p.m.) was measured as in Figure 1. Open symbols denote exposure without EGF and closed symbols with 10 ng ml$^{-1}$ EGF. In (**a**), (○ and ●) without, and (▽ and ▼) with 25 $\mu$M genistein, and in (**b**), (□ and ■) without, and (△ and ▲) with 50 $\mu$M tyrphostin-47.

**Figure 2** Effect of (**a**) genistein and (**b**) tyrphostin-47 on EGF-induced tyrosine phosphorylation in 2IHKE cells. Cells were incubated in the presence of genistein or tyrphostin-47 for 48 h at the indicated concentrations. Cells were then exposed to EGF (200 ng ml$^{-1}$ for 5 min). Cellular extracts were subjected to immunoblotting with a phosphotyrosine-specific antibody (see Materials and methods). 'EGF-R' denotes position of EGF receptor in immunoblots with an anti-EGF receptor antibody.

potent inhibitor of tyrosine kinase activity than tyrphostin-47. The effect of the inhibitors on DHA-stimulated proliferation of 2IHKE cells was studied. Genistein, at a concentration of 25 μM, inhibited proliferation and totally abolished the stimulatory effect of DHA in the 2IHKE cells, both in the absence and presence of EGF (Figure 3a). Under similar conditions, tyrphostin-47, at a concentration of 50 μM, also abolished stimulation of DNA synthesis by DHA, as shown in Figure 3b (tyrphostin-47 was not as inhibitory to [$^3$H]TdR incorporation as genistein). Densitometric scanning of the band corresponding to the EGF-R in Figure 2 revealed that the phosphotyrosine content at 25 μM genistein and 50 μM tyrphostin-47 was reduced to 32% and 43% of the controls respectively. Together, these data indicate the involvement of EGF receptor tyrosine kinase in the *n*-3 PUFA-induced stimulation of 2IHKE cell proliferation.

### *n-6 PUFAs exert an effect similar to n-3 PUFAs on DNA synthesis in 2IHKE cells*

The *n*-6 PUFAs LA and ARA were administered to 2IHKE cells under defined conditions similar to the *n*-3 fatty acid experiment (Figure 4). Low concentrations of either LA or ARA stimulated proliferation. In the absence of EGF, [$^3$H]TdR incorporation was significantly increased at 1–5 μM LA and 1–2.5 μM ARA respectively. Again, a synergistic effect of EGF was observed. In the case of LA and EGF a broader stimulatory concentration range (1–20 μM) was demonstrated, whereas [$^3$H]TdR incorporation was significantly increased at 1–2.5 μM ARA in the presence of EGF. As in the case of the *n*-3 PUFAs, high concentrations of LA or ARA inhibited cell proliferation.

### *Saturated fatty acids*

In contrast to the *n*-3 and *n*-6 PUFAs, the saturated fatty acids palmitic and stearic acid did not stimulate proliferation of 2IHKE cells at any concentrations tested (Figure 5). Rather, these fatty acids inhibited cell proliferation in a dose-dependent manner, PA being more inhibitory than SA.

### Discussion

We report here that low concentrations of *n*-3 PUFAs stimulated proliferation of the 2IHKE cell line under defined growth conditions. In the presence of EGF, the growth-stimulatory effect of PUFAs was even more profound. Specific tyrosine kinase inhibitors [genistein and tyrphostin-47 (Akiyama *et al.*, 1987; Gazit *et al.*, 1989)] inhibited EGF-induced protein tyrosine phosphorylation. In addition, genistein and tyrphostin-47 totally abrogated the growth-stimulatory effect of DHA, both in the absence or presence of EGF, suggesting interaction with tyrosine kinase signal transduction pathways especially involving the EGF-R. This is in agreement with the reported findings that an EGF-R-blocking antibody caused suppression of LA-stimulated proliferation of a human prostate cancer cell line (DU145M) in serum-free medium (Connolly and Rose, 1992). The possibility that other factors involved in the growth control of the 2IHKE cells may be affected by genistein and tyrphostin-47, however, cannot be excluded. *n*-6 and *n*-9 PUFAs have been reported to inhibit PDGF receptor tyrosine kinase activity in both intact cells and membrane preparations (Tomaska and Resnick, 1993). Treatment of the 2IHKE cells with *n*-3 PUFAs at growth-stimulatory conditions (in the absence of EGF) did not affect the EGF-induced level of cellular tyrosine phosphorylation (data not shown).

**Figure 4** Effect of *n*-6 PUFAs on [$^3$H]thymidine incorporation in 2IHKE cells. Cells were exposed to increasing concentrations of LA or ARA with and without EGF in SFM for 48 h. [$^3$H]TdR uptake (c.p.m.) was measured as in Figure 1. (○) LA without EGF, (●) LA with 10 ng ml$^{-1}$ EGF, (▽) ARA without EGF, and (▼) ARA with 10 ng ml$^{-1}$ EGF.

**Figure 5** Effect of saturated fatty acids on [$^3$H]thymidine incorporation in 2IHKE cells. Cells were exposed to increasing concentrations of PA or SA with and without EGF in SFM for 48 h. [$^3$H]TdR uptake (c.p.m.) was measured as in Figure 1. (○) PA without EGF, (●) PA with 10 ng ml$^{-1}$ EGF, (▽) SA without EGF, and (▼) SA with 10 ng ml$^{-1}$ EGF.

PUFAs have been shown to modulate EGF-mediated signal transduction (Bandyopadhyay *et al.*, 1987, 1993; Casabiell *et al.*, 1991). These studies included fatty acids of the *n*-6, *n*-7 and *n*-9 classes. However, studies on the action of *n*-3 PUFAs on signal transduction are limited, although modulation of the catalytic activity of protein kinase C and type I cAMP-dependent protein kinase has been demonstrated (Speizer *et al.*, 1991).

Interestingly, we observed a similar growth-stimulatory effect of low concentrations of both *n*-3 and *n*-6 PUFAs on 2IHKE cells, which have retained several phenotypical features of normal cells. Studies have shown that human prostate (PC-3) and breast (MDA-MB-231) cancer cell lines were growth stimulated by low concentrations of *n*-6 PUFA (LA) under defined conditions. However, in contrast to our results low concentrations of the *n*-3 PUFAs, EPA and DHA were growth inhibitory (Rose and Connolly, 1990, 1991). LA and ARA also stimulated cell proliferation of murine colon adenocarcinomas (Hussey and Tisdale, 1994). Perfusion of Morris hepatomas (7288CTC) *in situ* with donor whole blood containing added PUFAs resulted in an increased (*n*-6) or decreased (*n*-3) rate of DNA synthesis (Sauer and Dauchy, 1992). The increase in DNA synthesis rate was observed at high plasma concentrations of *n*-6 PUFAs (200–600 μM). *In vivo* studies have shown that PUFAs of the *n*-6 class may enhance both chemically induced and transplanted tumour development and that PUFAs of the *n*-3 class frequently exert an opposite effect, inhibiting tumour development (Roebuck *et al.*, 1981; Karmali *et al.*, 1984; Braden and Carroll, 1986; Borgeson *et al.*, 1989; Rose *et al.*, 1993; Mæhle *et al.*, 1995). The *in vivo* differences have been explained by differences in eicosanoid synthesis and different lipid peroxidation potential (Gonzalez *et al.*, 1993; Noguchi *et al.*, 1995). Our short-term studies on growth-regulatory control by PUFAs does not account for long-term *in vivo* effects, and a clear correlation between *in vivo* and *in vitro* data can therefore not be expected.

The stimulatory effect of the *n*-3 and *n*-6 PUFAs was confined to the 2IHKE cell line, which shows apparent normal EGF-mediated growth-regulatory control (unpublished data). In general, PUFAs have been implicated in promotion stages of carcinogenesis (Aylsworth *et al.*, 1984; Reddy *et al.*, 1991; Ronai *et al.*, 1991). Our data on the action of low concentrations of PUFAs are consistent with a multistep carcinogenesis model where stimulation of clonogenic growth of initiated cells (2IHKE) will enhance the possibility of a hit by secondary carcinogens, and this in turn may result in tumorigenic conversion. The growth-stimulatory effect of PUFAs correlates with normal EGF growth-regulatory control. In our *in vitro* model, the 1IHKE and 1THKE*ras* cell lines showed a marked loss of stimulatory response to low concentrations of PUFAs, indicating an alteration taking place during the *in vitro* carcinogenic process. These results correlate with our previous observations that the 1IHKE and 1THKE*ras* cell lines have abrogated normal growth-regulatory control in respect of mitogenic response to EGF and expression of the EGF-R (Mollerup *et al.*, 1996). The proliferation of normal cells is a highly regulated process, controlled by the interplay of growth-inducing and growth-inhibitory signals. Although normal human kidney epithelial cells are stimulated by PUFAs *in vitro* (data not shown), this characteristic might not be expressed *in vivo*, indicating no conflict with the perception of PUFAs functioning as tumour promoters.

Using defined growth conditions, we observed an increased resistance towards the growth-inhibitory effect of high concentrations of DHA with increased transformation/malignant potential of the human kidney epithelial cell lines (2IHKE < 1IHKE < 1THKE*ras*). Sensitivity to the growth-inhibitory effect of PUFAs varies considerably between different cell types (Krokan *et al.*, 1993; Mæhle *et al.*, 1995). In contrast to the data presented here, it has been reported that normal cells may be more resistant to the inhibitory effect than tumour cells *in vitro* (Begin *et al.*, 1986; Krokan *et al.*, 1993), and it might be expected that sensitivity should increase during development of the transformed phenotype within cell lines with a common origin.

In conclusion, we have shown that *n*-3 and *n*-6 fatty acids may stimulate the proliferation of immortalised human kidney epithelial cells with apparent normal EGF growth-regulatory control. This effect apparently involves EGF-R tyrosine kinase activity. Furthermore, our results demonstrate that the stimulatory growth response to the PUFAs was abrogated during *in vitro* transformation of the cell lines. Further studies are needed to determine the specific mechanism(s) involved.

### Abbreviations

EGF, epidermal growth factor; EGF-R, epidermal growth factor receptor; PA, palmitic acid (C16:0); SA, stearic acid (C18:0); LA, linoleic acid (C18:2, *n*-6); ARA, arachidonic acid (C20:4, *n*-6); EPA, eicosapentaenoic acid (C20:5, *n*-3); DHA, docosahexaenoic acid (C22:6, *n*-3); PUFA, polyunsaturated fatty acid; TdR, thymidine.

### Acknowledgements

The authors wish to acknowledge Dr Edgar Rivedal, The Norwegian Radium Hospital, for constructive discussion of the manuscript. This study was supported by Pronova as, Norway and the EU programme AIR CT93-0860.

### References

AKIYAMA T, ISHIDA J, NAKAGAWA S, OGAWARA H, WATANABE S-I, ITOH N, SHIBUYA M AND FUKAMI Y. (1987). Genistein, a specific inhibitor of tyrosine-specific protein kinases. *J. Biol. Chem.*, **262**, 5592–5595.

AYLSWORTH CF, JONE C, TROSKO JE, MEITES J AND WELSCH CW. (1984). Promotion of 7,12-dimethylbenz[a]anthracene-induced mammary tumorigenesis by high dietary fat in the rat: possible role of intercellular communication. *J. Natl Cancer Inst.*, **72**, 637–645.

BANDYOPADHYAY GK, IMAGAWA W, WALLACE D AND NANDI S. (1987). Linoleate metabolites enhance the *in vitro* proliferative response of mouse mammary epithelial cells to epidermal growth factor. *J. Biol. Chem.*, **262**, 2750–2756.

BANDYOPADHYAY GK, HWANG S-I, IMAGAWA W AND NANDI S. (1993). Role of polyunsaturated fatty acids as signal transducers: Amplification of signals from growth factor receptors by fatty acids in mammary epithelial cells. *Prost. Leuk. Ess. Fatty Acids*, **48**, 71–78.

BEGIN ME. (1989). Tumor cytotoxicity of essential fatty acids. *Nutrition*, **5**, 258–260.

BEGIN ME, ELLS G, DAS UN AND HORROBIN DF. (1986). Differential killing of human carcinoma cells supplemented with *n*-3 and *n*-6 polyunsaturated fatty acids. *J. Natl Cancer Inst.*, **77**, 1053–1062.

BORGESON CE, PARDINI L, PARDINI RS AND REITZ RC. (1989). Effects of dietary fish oil on human mammary carcinoma and on lipid-metabolizing enzymes. *Lipids*, *24*, 290–295.

BRADEN LM AND CARROLL KK. (1986). Dietary polyunsaturated fat in relation mammary carcinogenesis in rat. *Lipids*, **21**, 285–288.

CARTER CA, MILHOLLAND RJ, SHEA W AND IP MM. (1983). Effect of the prostaglandin synthase inhibitor indomethacin on 7,12-dimethylbenz (a) anthracene-induced mammary tumorigenesis in rats fed different levels of fat. *Cancer Res.*, **43**, 3559–3562.

CASABIELL X, PANDIELLA A AND CASANUEVA FF. (1991). Regulation of epidermal-growth-factor-receptor signal transduction by *cis*-unsaturated fatty acids. Evidence for a protein kinase C-independent mechanism. *Biochem. J.*, **287**, 679–687.

CAVE WT. (1991). Dietary *n*-3 ($\omega$-3) polyunsaturated fatty acid effects on animal tumorigenesis. *FASEB J.*, **5**, 2160–2166.

CONNOLLY JM AND ROSE DP. (1992). Interaction between epidermal growth factor-mediated autocrine regulation and linoleic acid-stimulated growth of a human prostate cancer cell line. *Prostate*, *20*, 151–158.

COREY EJ, SHIH C AND CASHMAN JR. (1983). Docosahexaenoic acid is a strong inhibitor of prostaglandin but not leukotrine biosynthesis. *Proc. Natl Acad. Sci. USA*, **80**, 3581–3584.

CULP BR, TITUS BJ AND LANDS WE. (1979). Inhibition of prostaglandin biosynthesis by eicosapentaenoic acid. *Prostaglandins Med.*, **3**, 269–278.

DE BRAVO MG, DE ANTUENO RJ, TOLEDO J, DE TOMAS ME, MERCURI OF AND QUINTANS C. (1991). Effects of an eicosapentaenoic and docasahexaenoic acid concentrate on a human lung carcinoma grown in nude mice. *Lipids*, **26**, 866–870.

DISTEL RJ, ROBINSON GS AND SPIEGELMAN BM. (1992). Fatty acid regulation of gene expression. Transcriptional and post-transcriptional mechanisms. *J. Biol. Chem.*, **267**, 5937–5941.

FAZIO VM, BARRERA G, MARTINOTTI S, FARACE MG, GIGLIONI B, FRATI L, MANZARI V AND DIANZANI MU. (1992). 4-Hydroxynonenal, a product of cellular lipid peroxidation, which modulates c-*myc* and globin gene expression in K562 erythroleukemic cells. Cancer Res., **52**, 4866–4871.

GAZIT A, YAISH P, GILON C, AND LEVITZKI A. (1989). Tyrphostins I: synthesis and biological activity of protein tyrosine kinase inhibitors. *J. Med. Chem.*, **32**, 2344–2352.

GLASGOW WC, AFSHARI CA, BARRETT JC AND ELING TE. (1992). Modulation of the epidermal growth factor mitogenic response by metabolites of linoleic and arachidonic acid in Syrian hamster embryo fibroblasts. *J. Biol. Chem.*, **267**, 10771–10779.
GONZALEZ MJ. (1992). Lipid peroxidation and tumor growth: an inverse relationship. *Med. Hypotheses*, **38**, 106–110.
GONZALEZ MJ, SCHEMMEL RA, DUGAN L-R JR, GRAY JI AND WELSCH CW. (1993). Dietary fish oil inhibits human breast carcinoma growth: a function of increased lipid peroxidation. *Lipids*, **28**, 827–832.
HAUGEN A, RYBERG D, HANSTEEN I-L AND AMSTAD P. (1990). Neoplastic transformation of a human kidney epithelial cell line with v-Ha-*ras* oncogene. *Int. J. Cancer*, **45**, 572–577.
HOWE CR, FRIEDENREICH CM, JAIN M AND MILLER AB. (1991). A cohort study of fat intake and risk of breast cancer. *J. Natl Cancer Inst.*, **83**, 336–340.
HUSSEY HJ AND TISDALE MJ. (1994). Effect of polyunsaturated fatty acids on the growth of murine colon adenocarcinomas *in vitro* and *in vivo*. *Br. J. Cancer*, **70**, 6–10.
HØSTMARK AT AND LYSTAD E. (1992). Growth inhibition of human hepatoma cells (HepG2) by polyunsaturated fatty acids. Protection by albumin and vitamin E. *Acta Physiol. Scand.*, **144**, 83–88.
KAIZER L, BOYD NF, KRIUKOV V AND TRITCHLER D. (1989). Fish consumption and breast cancer risk: an ecological study. *Nutr. Cancer*, **12**, 61–68.
KARMALLI RA, MARSH J AND FUCHS C. (1984). Effect of omega-3 fatty acids on growth of a rat mammary tumor. *J. Natl Cancer Inst.*, **73**, 457–461.
KARMALI RA. (1987). Eicosanoids in neoplasia. *Prev. Med.*, **16**, 493–502.
KROKAN HE, RUDRA PK, SCHØBERG S, SLETTAHJELL W, SOLUM K, MOLLERUP S, EILERTSEN E, MÆHLE L AND HAUGEN A. (1993). Effect of n-3 fatty acids on the growth of human tumor cell lines in cell culture and in nude mice. In *Omega-3 Fatty Acids: Metabolism and Biological Effects*. Drevon CA, Baksaas I and Krokan HE. (eds) pp. 327–334. Birkhäuser Verlag: Basle.
LAEMMLI UK. (1970). Cleavage of structural proteins during the assembly of the head of bacteriophage T4. *Nature*, **227**, 680–685.
LOWRY OH, ROSEBROUGH NJ, FARR AL AND RANDALL RJ. (1951). Protein measurement with the folin phenol reagent. *J. Biol. Chem.*, **193**, 265–275.
MÆHLE L, METCALF RA, RYBERG D, BENNETT WP, HARRIS CC AND HAUGEN A. (1992). Altered p53 gene structure and expression in human epithelial cells after exposure to nickel. *Cancer Res.*, **52**, 218–221.
MÆHLE L, EILERTSEN E, MOLLERUP S, SCHØNBERG S, KROKAN HE AND HAUGEN A. (1995). Effect of n-3 fatty acids during neoplastic progression and comparison of *in vitro* and *in vivo* sensitivity of two human tumour cell lines. *Br. J. Cancer*, **71**, 691–696.
MILLER AB, HOWE GR, JAIN M, CRAIB KJP AND HARRISON L. (1983). Food items and food groups as risk factors in a case–control study of diet and colorectal cancer. *Int. J. Cancer*, **32**, 155–161.
MOLLERUP S, RIVEDAL E, MÆHLE L AND HAUGEN A. (1996). Nickel(II) induces alterations in EGF- and TFG-$\beta_1$- mediated growth control during malignant transformation of human kidney epithelial cells. *Carcinogenesis*, **17**, 361–367.
MORISAKI N, SPRECHER H, MILO GE AND CORNWELL DG. (1992). Fatty acid specificity in the inhibition of cell proliferation and its relationship to lipid peroxidation and prostaglandin biosynthesis. *Lipids*, **17**, 893 – 899.
NICHOLSON ML, NEOPTOLEMOS JP, CLAYTON HA AND HEAGERTY AM. (1988). Diet and colorectal cancer. *Int. Clin. Nutr. Rev.*, **8**, 180–197.
NOGUCHI M, KITAGAWA H, MIYAZAKI I AND MIZUKAMI Y. (1993). Influence of esculetin on incidence, proliferation, and cell kinetics of mammary carcinomas induced by 7,12-dimethylbenz[a]anthracene in rats on high- and low-fat diets. *Jpn. J. Cancer Res.*, **84**, 1010–1014.
NOGUCHI M, ROSE DP, EARASHI M AND MIYAZAKI I. (1995). The role of fatty acids and eicosanoid synthesis inhibitors in breast carcinoma. *Oncology*, **52**, 265–271.
O'CONNOR TP, ROEBUCK BD, PETERSON FJ, LOKESH B, KINSELLA JE AND CAMBELL TC. (1989). Effect of dietary omega-3 and omega-6 fatty acids on development of azaserine-induced preneoplastic lesions in rat pancreas. *J. Natl Cancer Inst.*, **81**, 858–863.
REDDY BS AND MARUYAMA H. (1986). Effect of different levels of dietary corn oil and lard during the initiation phase of colon carcinogenesis in F344 rats. *J. Natl Cancer Inst.*, **77**, 815–822.
REDDY BS, BURILL C AND RIGOTTI J. (1991). Effects of diets high in Ω-3 and Ω-6 fatty acids on initiation and postinitiation stages of colon carcinogenesis. *Cancer Res.*, **51**, 487–491.
ROEBUCK BD, YAGER JDJ, LONGNECKER DS AND WILPONE SA. (1981). Promotion by unsaturated fat of azaserine-induced pancreatic carcinogenesis in the rat. *Cancer Res.*, **41**, 3961–3966.
RONAI Z, LAU Y AND COHEN LA. (1991). Dietary N-3 fatty acids do not affect induction of Ha-*ras* mutations in mammary glands of NMU-treated rats. *Mol. Carcinogen.*, **4**, 120–128.
ROSE DP AND CONNOLLY JM. (1990). Effects of fatty acids and inhibitors of eicosanoid synthesis on the growth of a human breast cancer cell line in culture. *Cancer Res.*, **50**, 7139–7144.
ROSE DP AND CONNOLLY JM. (1991). Effects of fatty acids and eicosanoid synthesis inhibitors on the growth of two human prostate cancer cell lines. *Prostate*, **18**, 243–254.
ROSE DP AND CONNOLLY JM. (1993). Effects of dietary omega-3 fatty acids on human breast cancer growth and metastases in nude mice. *J. Natl Cancer Inst.*, **85**, 1743–1747.
ROSE DP, HATALA MA, CONNOLLY JM AND RAYBURN J. (1993). Effect of diets containing different levels of linoleic acid on human breast cancer growth and lung metastasis in nude mice. *Cancer Res.*, **53**, 4686–4690.
SAUER LA AND DAUCHY RT. (1992). The effect of omega-6 and omega-3 fatty acids on $^3$H-thymidine incorporation in hepatoma 7288CTC perfused *in situ*. *Br. J. Cancer*, **66**, 297–303.
SPEIZER LA, WATSON MJ AND BRUNTON LL. (1991). Differential effects of omega-3 fish oils on protein kinase activities *in vitro*. *Am. J. Physiol.*, **261**, E109–E114.
TIWARI RK, MUKHOPADHYAY B, TELANG NT AND OSBORNE MP. (1991). Modulation of gene expression by selected fatty acids in human breast cancer cells. *Anticancer Res.*, **11**, 1383–1388.
TOMASKA L AND RESNICK RJ. (1993). Suppression of platelet-derived growth factor receptor tyrosine kinase activity by unsaturated fatty acids. *J. Biol. Chem.*, **268**, 5317–5322.
TVEITO G, HANSTEEN I-L, DALEN H AND HAUGEN A. (1989). Immortalization of normal human kidney epithelial cells by nickel(II). *Cancer Res.*, **49**, 1829–1835.
ULLRICH A AND SCHLESSINGER J. (1990). Signal transduction by receptors with tyrosine kinase activity. *Cell*, **61**, 203–212.
VAN DER GEER P AND HUNTER T. (1994). Receptor protein-tyrosine kinases and their signal transduction pathways. *Ann. Rev. Cell. Biol.*, **10**, 251–337.